# ACEI/ARB Medication During ICU Stay Decrease All-Cause In-hospital Mortality in Critically Ill Patients With Hypertension: A Retrospective Cohort Study Based on Machine Learning

**DOI:** 10.3389/fcvm.2021.787740

**Published:** 2022-01-12

**Authors:** Boshen Yang, Sixuan Xu, Di Wang, Yu Chen, Zhenfa Zhou, Chengxing Shen

**Affiliations:** ^1^Department of Cardiology, Shanghai Jiao Tong University Affiliated Sixth People's Hospital, Shanghai, China; ^2^Intelligent Transportation Systems Research Center, School of Transportation, Southeast University, Nanjing, China

**Keywords:** hypertension, intensive care units, ACEI/ARB, prognosis predictors, machine learning

## Abstract

**Background:** Hypertension is a rather common comorbidity among critically ill patients and hospital mortality might be higher among critically ill patients with hypertension (SBP ≥ 140 mmHg and/or DBP ≥ 90 mmHg). This study aimed to explore the association between ACEI/ARB medication during ICU stay and all-cause in-hospital mortality in these patients.

**Methods:** A retrospective cohort study was conducted based on data from Medical Information Mart for Intensive Care IV (MIMIC-IV) database, which consisted of more than 40,000 patients in ICU between 2008 and 2019 at Beth Israel Deaconess Medical Center. Adults diagnosed with hypertension on admission and those had high blood pressure (SBP ≥ 140 mmHg and/or DBP ≥ 90 mmHg) during ICU stay were included. The primary outcome was all-cause in-hospital mortality. Patients were divided into ACEI/ARB treated and non-treated group during ICU stay. Propensity score matching (PSM) was used to adjust potential confounders. Nine machine learning models were developed and validated based on 37 clinical and laboratory features of all patients. The model with the best performance was selected based on area under the receiver operating characteristic curve (AUC) followed by 5-fold cross-validation. After hyperparameter optimization using Grid and random hyperparameter search, a final LightGBM model was developed, and Shapley Additive exPlanations (SHAP) values were calculated to evaluate feature importance of each feature. The features closely associated with hospital mortality were presented as significant features.

**Results:** A total of 15,352 patients were enrolled in this study, among whom 5,193 (33.8%) patients were treated with ACEI/ARB. A significantly lower all-cause in-hospital mortality was observed among patients treated with ACEI/ARB (3.9 vs. 12.7%) as well as a lower 28-day mortality (3.6 vs. 12.2%). The outcome remained consistent after propensity score matching. Among nine machine learning models, the LightGBM model had the highest AUC = 0.9935. The SHAP plot was employed to make the model interpretable based on LightGBM model after hyperparameter optimization, showing that ACEI/ARB use was among the top five significant features, which were associated with hospital mortality.

**Conclusions:** The use of ACEI/ARB in critically ill patients with hypertension during ICU stay is related to lower all-cause in-hospital mortality, which was independently associated with increased survival in a large and heterogeneous cohort of critically ill hypertensive patients with or without kidney dysfunction.

## Introduction

Hypertension is the leading risk factor for cardiovascular disease and all-cause mortality over the world (1, 2). The prevalence of hypertension with SBP ≥ 140 mmHg and/or DBP ≥ 90 mmHg was around 31.1% among the global adult population (1.39 billion people) in 2010 (3). Pressure overload caused by hypertension could promote cardiac remodeling, and is responsible for the development of arrhythmia, heart failure and coronary heart disease (4), kidney disease as well as cerebral diseases, such as stroke and cerebral hemorrhage (5). All above-mentioned diseases are common comorbidities in patients who are admitted to intensive care units (ICU). The reported ICU mortality was 11.3% in 1996 and 12.0% in 2010 (6). The top priority right now is to find the risk factors for high ICU mortality and controlling hypertension might be one strategy to effectively reduce the ICU mortality in critically ill patients with high blood pressure during ICU stay.

Multiple hypertension medications with confirmed effects and limited side effects are now available, including β-blocker, diuretics, angiotensin-converting enzyme inhibitor (ACEI), angiotensin receptor blocker (ARB), and calcium channel blocker (CCB) as well as others. ACEI was considered more effective in reducing small artery remodeling (7), large artery stiffness (8), and left ventricular hypertrophy (9) compared to CCB, diuretic and β-blocker, though these drugs had similar efficacy on lowering peripheral blood pressure. Like ACEI, the use of ARB was also associated with improvement of endothelial function, reduction of large artery stiffening, and left ventricular hypertrophy (LVH) (10–12). Both ACEI and ARB target on the renin-angiotensin- aldosterone system (RAAS) with similar hemodynamic effects. A large-scale cohort study reported that a small population with increased creatinine levels posts ACEI/ARB use may face an increased risk of worse long-term outcomes (13). Among critically ill patients treated in ICU, the overall incidence of acute kidney injury (AKI) ranged from 20 to 50% and the risk of mortality was significantly higher among AKI patients (14). The impact of ACEI/ARB use on the prognosis of critically ill patients with hypertension, especially in patients with chronic or acute kidney injury, needs further exploration.

Recently, with the rapid development of artificial intelligence, machine learning (ML) is increasingly used in the field of medicine, also in predicting mortality. The aim of this study is thus to investigate whether the use of ACEI/ARB during ICU stay was associated with clinical outcomes among critically ill patients with hypertension in the absence or presence of chronic or acute kidney injury using PSM and machine learning models.

## Methods

### Data Source

The data were from a freely accessible critical care database, the Medical Information Mart for Intensive Care IV (MIMIC-IV) database, which consisted of more than 40,000 patients in ICU between 2008 and 2019 at Beth Israel Deaconess Medical Center (15). We have passed the “protecting human subjects training” and obtained a certificate to access the database. The institutional review boards of Beth Israel Deaconess Medical Center and the Massachusetts Institute of Technology approved the establishment of this database. All participants of this study were anonymous. Thus, no informed consent and ethical approval statement were required for this article. Variables with more than 30% missing values were excluded. Otherwise, other missing values were filled using multiple imputation method conducted in Stata software version 14.0. We conducted Pearson correlation test to confirm that there is no significant correlation between some unrelated variables to ensure the reliability of the data (Supplementary Figure 1).

### Study Population and Study Design

**Figure 1:**
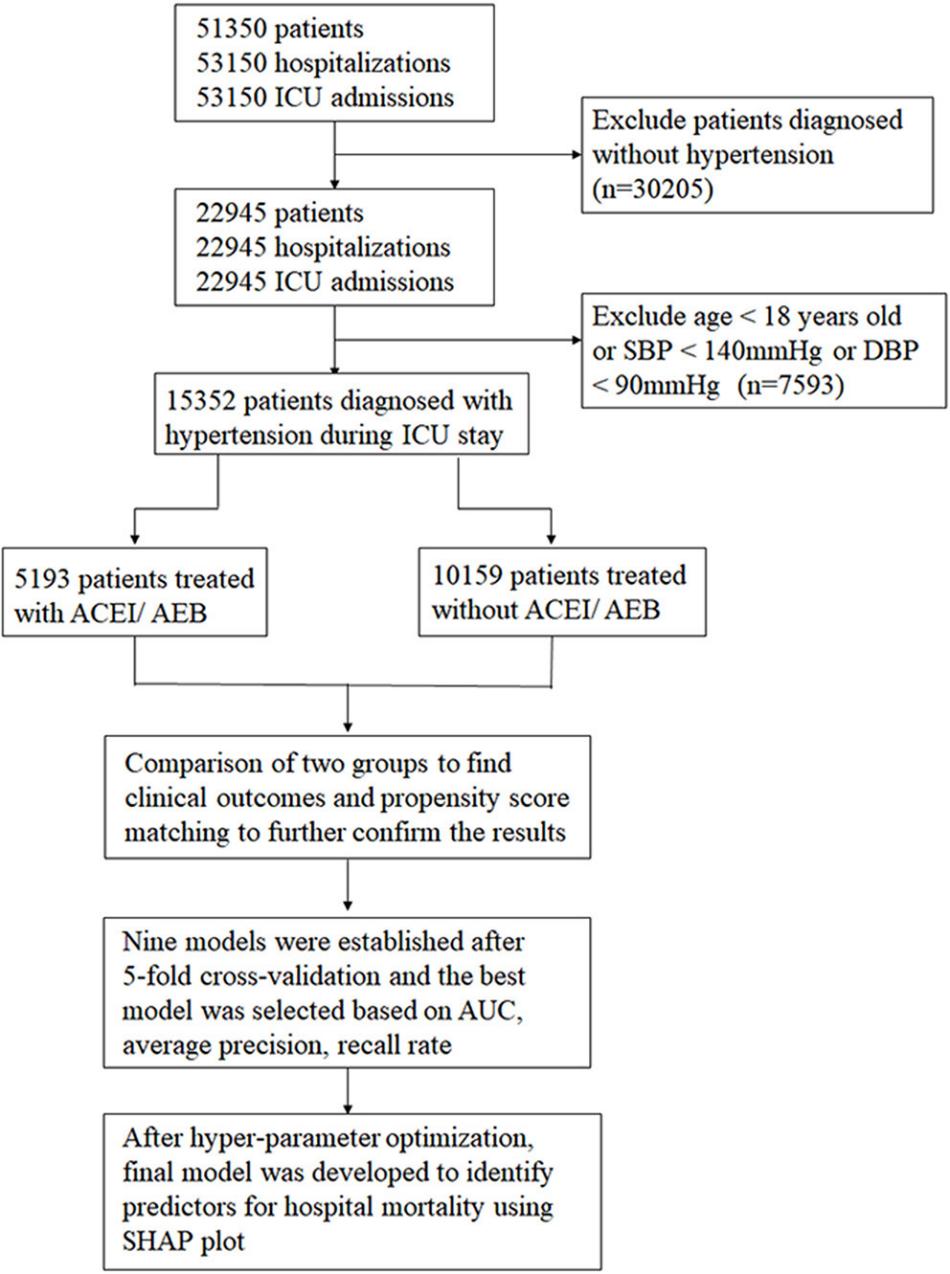
Study protocol flowchart.

This is a retrospective cohort study in a large and heterogeneous cohort of critically ill patients with hypertension. The patients aged ≥18 years were enrolled as our study participants. All these patients were diagnosed with hypertension on admission, while those with malignant hypertension and pregnancy-related hypertension were excluded. First, we identified all hospitalized patients with ICU stay. After entering the ICU, patients with the first systolic blood pressure measurement < 140 mmHg and/or DBP < 90 mmHg were excluded to avoid hypotension caused by other pathological factors and these people might not need antihypertensive drug treatment during ICU stay. For patients with more than one ICU stay, only the first ICU admission in the first hospitalization was included. Hypertensive patients were divided into three stages: stage 1 hypertension (140 ≤ BP < 160 and/or 90 ≤ DBP <100), stage 2 hypertension (160 ≤ SBP < 180 and/or 100 ≤ DBP < 110), stage 3 hypertension (SBP ≥ 180 and/or DBP ≥ 110). The detailed research process is displayed in Figure 1.

### Outcome Variables and Definitions

All-cause in-hospital mortality was extracted as the primary outcome, which was defined as death during hospitalization. The secondary endpoints included 28-day mortality (after ICU admission), ICU mortality, length of ICU stay, length of hospital stay. Maximum sequential organ failure assessment (SOFA) score during ICU stay was also chosen as the secondary endpoint. For patients with more than one hospital admission and more than one ICU stay during one hospitalization, hospital/ICU mortality and hospital/ICU stay were determined only by the first ICU stay in the first hospital admission.

### Development of ML Models and Model Explainability

Nine ML models were employed in this study: Logistic regression, Support Vector Classifier (SVC), Decision Tree, Bagging, Gradient Boosting Machine (GBM), K-nearest neighbors (KNN), Random Forest, XGBoost, and LightGBM. A sample of 80% of the cohort generated randomly using a seed was employed for the training set while the remaining 20% was used for testing. For training and tuning of the models, each model was validated by 5-fold cross-validation and the average accuracy was calculated. Areas under the receiver operating characteristic curves (AUCs) were used to evaluate the performance of models as well as the precision and recall rate. Among them, the model with the best performance was then adjusted for optimal hyper-parameters using Grid and random hyper-parameter search for further optimization. And the same input variables were supplied in the final model to predict the hospital mortality of critically ill patients with hypertension. These models are the most commonly used for binary classification problems in predicting mortality.

To identify potentially relevant features for hospital mortality of the study participants and make the model interpretable, the Shapley additive explanation (SHAP) was used in this study. This method assessed the importance of each feature using a game-theoretic approach. The SHAP plot was drawn to show key features with highest feature importance.

### Statistical Analysis

Data are presented in the tables according to different types and distributions of variables. Continuous variables were presented as mean ± standard deviation or median (25–75 percentiles), which were tested by Student's *t*-test or Wilcoxon rank-sum test. Categorical variables were presented as numbers (percentages) and tested by Chi-square (or Fisher's exact) tests. The Kaplan-Meier method and log-rank tests were used to compare the survival distributions among patients treated with or without ACEI/ARB.

Patients were categorized as treated with ACEI/ARB or not during ICU stay, and a propensity score for each patient was calculated using multi-variable logistic regression models. The cases were matched to the nearest-neighbor controls at a 1:1 ratio based on their propensity scores. After matching, the baseline characteristics of the two groups were compared using univariate analyses mentioned above and all of their *P*-values were higher than 0.05. All statistical analysis was performed using the software STATA software version 14.0 and SPSS software version 23.0. An alpha level of 0.05 was set for statistical significance.

## Results

### Baseline Characteristics of the Study Population

After the selection of inclusion and exclusion criteria, a total of 15,352 patients with hypertension in ICU were included in the study population. The baseline characteristics of the two groups are displayed in Table 1. Among them, 5,193 (33.8%) were treated with ACEI/ARB, while 10,159 (66.2%) were treated without AECI/ARB. The average age of the total study participants was 66.23 ± 17.4. Patients treated with ACEI/ARB were older than those treated without ACEI/ARB (69.8 ± 18.5 vs. 64.4 ± 18.4), accompanying with higher SBP [152 (143–163) vs. 148 (141–158)]. The DBP values were higher in patients treated without ACEI/ARB [86 (73–96) vs. 82 (69–95)] as well as the heart rate [84 (73–96) vs. 80 (70–91)]. As for comorbidities, patients treated with ACEI/ARB had a higher incidence of congestive heart failure (27.1 vs. 15.8%), coronary heart disease (33.6 vs. 18.7%), arrhythmia (27.3 vs. 22.3%), cardiac shock (2.2 vs. 1.3%), diabetes (40.6 vs. 25.7%), cerebral hemorrhage (9.7 vs. 5.3%), CKD (20.9 vs. 18.2%). With regard to laboratory events, BUN [19.0 (13.7–27.5) vs. 17.9 (12–29.7)], RBC [3.7 (3.3–4.2) vs. 3.6 (3.1–4.2)], Hemoglobin [11.1 (9.7–12.6) vs. 10.9 (9.4–12.5)], Creatinine [0.95 (0.74–1.30) vs. 0.90 (0.70–1.40)], PLT [206.0 (159.8–261.5) vs. 200.3 (146.9–262.0)], Calcium [8.5 (8.1–8.9) vs. 8.6 (8.2–9.0)], Bicarbonate [24.3 (22.3–26.5) vs. 24.0 (21.5–26.0)], Glucose [128.3 (109.0–160.5) vs. 124.0 (105.5–152.0)], while WBC [9.8 (7.6–12.6) vs. 10.0 (7.5–13.4)] and Chloride [103.7 (100.5–106.6) vs. 104.0 (101.0–107.0)] were lower than those treated without ACEI/ARB. No statistically significant differences were observed in gender, COPD, hypothyroidism, cerebral infarction, sodium or potassium. Besides, patients treated with ACEI/ARB had higher hypertension stages and higher frequency of β-blocker use (63.0 vs. 42.0%), CCB use (31.6 vs. 18.2%), diuretics use (36.1 vs. 27.5%), and intravenous antihypertensive drugs (15.2 vs. 7.0%).

**Table 1:** Comparison of baseline characteristics between patients treated with ACEI/ARB and those treated without ACEI/ARB.

**Variables**	**All patience (*n* = 15,352)**	**ACEI/ARB (*n* = 5,193)**	**No-use of ACEI/ARB (*n* = 10,159)**	***P*-value**
Age (years)	66.23 ± 17.4	69.8 ± 18.5	64.4 ± 18.4	<0.001
Males [*n* (%)]	8,493 (55.3)	2,821 (54.3)	5,672 (55.8)	0.075
Heart rate (/min)	83 (72–94)	80 (70–91)	84 (73–96)	<0.001
**Blood pressure**				
SBP (mmHg)	149 (142–159)	152 (143–163)	148 (141–158)	<0.001
DBP (mmHg)	85 (71–96)	82 (69–95)	86 (73–96)	<0.001
**Comorbidity**				
Congestive heart failure [*n* (%)]	3,012 (19.6)	1,406 (27.1)	1,606 (15.8)	<0.001
Coronary heart disease [*n* (%)]	3,641 (23.7)	1,745 (33.6)	1,896 (18.7)	<0.001
Arrhythmia [*n* (%)]	3,684 (24.0)	1,418 (27.3)	2,266 (22.3)	<0.001
Cardiac shock [*n* (%)]	251 (1.6)	114 (2.2)	137 (1.3)	<0.001
Valvular disease [*n* (%)]	1,529 (10.0)	681 (13.1)	848 (8.3)	<0.001
COPD [*n* (%)]	1,249 (8.1)	419 (8.1)	830 (8.2)	0.828
Respiratory failure [*n* (%)]	3,481 (22.7)	1,063 (20.5)	2,418 (23.8)	<0.001
Diabetes [*n* (%)]	4,721 (30.8)	2,109 (40.6)	2,612 (25.7)	<0.001
Hypothyroidism [*n* (%)]	1,915 (12.5)	667 (12.8)	1,248 (12.3)	0.321
Cerebral hemorrhage [*n* (%)]	1,042 (6.8)	505 (9.7)	537 (5.3)	<0.001
Cerebral infarction [*n* (%)]	545 (3.6)	196 (3.8)	349 (3.4)	0.283
AKI [*n* (%)]	3,980 (12.9)	1,296 (25.0)	2,684 (26.4)	0.05
CKD [*n* (%)]	2,929 (19.1)	1,084 (20.9)	1,845 (18.2)	<0.001
Lymphoma [*n* (%)]	159 (1.0)	28 (0.5)	131 (1.3)	<0.001
Anemia [*n* (%)]	2,686 (17.5)	769 (14.8)	1,917 (18.9)	<0.001
**Laboratory events**				
WBC (K/uL)	10.0 (7.5–13.1)	9.8 (7.6–12.6)	10.0 (7.5–13.4)	0.001
BUN (mg/dL)	18.0 (12.5–30.0)	19.0 (13.7–27.5)	17.9 (12–29.7)	<0.001
RBC (m/uL)	3.7 (3.2–4.2)	3.7 (3.3–4.2)	3.6 (3.1–4.2)	<0.001
Hemoglobin (g/dL)	11.0 (9.5–12.5)	11.1 (9.7–12.6)	10.9 (9.4–12.5)	<0.001
Creatinine (mg/dL)	0.90 (0.7–1.4)	0.95 (0.74–1.30)	0.90 (0.70–1.40)	<0.001
PLT (K/uL)	202.6 (151.7–261.8)	206.0 (159.8–261.5)	200.3 (146.9–262.0)	<0.001
Sodium (mmol/L)	139.0 (136.7–141.5)	139.0 (136.8–141.5)	139.0 (136.7–141.5)	0.569
potassium (mmol/L)	4.0 (3.8–4.3)	4.0 (3.8–4.3)	4.0 (3.8–4.4)	0.158
calcium (mmol/L)	8.5 (8.1–8.9)	8.6 (8.2–9.0)	8.5 (8.1–8.9)	<0.001
Bicarbonate (mmol/L)	24.0 (22.0–26.2)	24.3 (22.3–26.5)	24.0 (21.5–26.0)	<0.001
Chloride (mmol/L)	104.0 (100.8–107.0)	103.7 (100.5–106.6)	104.0 (101.0–107.0)	<0.001
Glucose (mg/dL)	125.5 (107.0–155.0)	128.3 (109.0–160.5)	124.0 (105.5–152.0)	<0.001
**Hypertension stage**				
Stage 1 hypertension [*n* (%)]	9,698 (63.2)	3,047 (58.7)	6,651 (65.5)	<0.001
Stage2 hypertension [*n* (%)]	3,601 (23.5)	1,360 (26.2)	2,241 (22.1)	<0.001
Stage3 hypertension [*n* (%)]	2,053 (13.4)	786 (15.1)	1,267 (12.5)	<0.001
**Drugs**				
β blocker [*n* (%)]	7,542 (49.1)	3,274 (63.0)	4,268 (42.0)	<0.001
Calcium channel blocker [*n* (%)]	3,490 (25.7)	1,643 (31.6)	1,847 (18.2)	<0.001
Diuretics [*n* (%)]	4,671 (30.4)	1,875 (36.1)	2,796 (27.5)	<0.001
Intravenous drugs [*n* (%)]	1,503 (9.8)	790 (15.2)	713 (7.0)	<0.001

### Clinical Outcomes of the Two Comparing Groups

**Figure 2:**
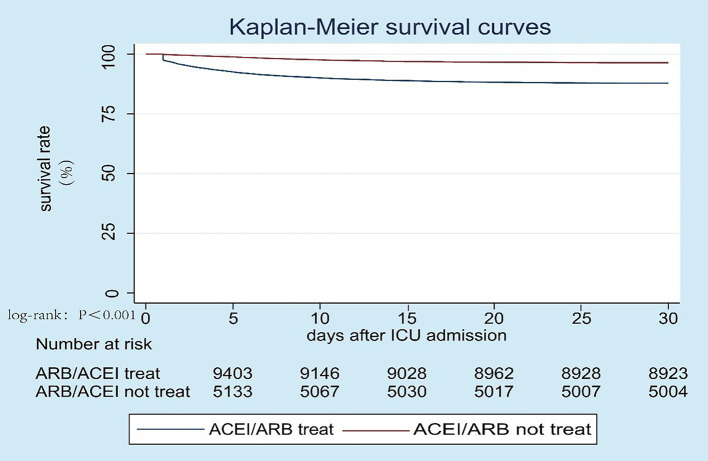
Kaplan-Meier survival curve between patients treated with ACEI/ARB and those without ACEI/ARB, log-rank test: *P* < 0.001.

Unadjusted clinical outcomes by the comparison of the two groups are shown in Table 2. In total, 1,492 patients (9.7%) died during hospitalization, while hospital mortality was significantly higher in patients treated without ACEI/ARB (12.7 vs. 3.9%) compared to those treated with ACEI/ARB. Similar results were found for 28-day mortality (12.2 vs. 3.6%) and ICU mortality (8.1 vs. 1.8%). As for the length of stay in hospital and ICU, the median lengths of hospital LOS and ICU LOS were close, while the statistical differences were significant. And patients treated without ACEI/ARB had higher SOFA [4 (2–6) vs. 3 (1–5)] score than those treated with ACEI/ARB. The unadjusted survival curve for patients with different ACEI/ARB use was shown in a Kaplan-Meier plot in Figure 2 (log-rank test: *P* < 0.001).

**Table 2:** Unadjusted outcomes in critically ill patients with hypertension treated with or without ACEI/ARB.

**Variables**	**Total (*n* = 15,352)**	**ARB/ACEI (*n* = 5,193)**	**No-use of ACEI/ARB (*n* = 10,159)**	***P*-value**
Hospital mortality [*n* (%)]	1,492 (9.7)	205 (3.9)	1,287 (12.7)	<0.001
Day mortality [*n* (%)]	1,425 (9.3)	189 (3.6)	1,236 (12.2)	<0.001
ICU mortality [*n* (%)]	914 (6.0)	91 (1.8)	823 (8.1)	<0.001
ICU LOS (days)	1.87 (1.02–3.61)	1.92 (1.06–3.73)	1.85 (0.99–3.55)	0.001
Hospital LOS (days)	6.25 (3.58–10.96)	6.58 (3.88–10.96)	6.04 (3.29–11)	<0.001
SOFA score	3 (2–6)	3 (1–5)	4 (2–6)	<0.001

### The Baseline Characteristics and Clinical Outcomes After Propensity Score Matching

As shown in Table 3, the cases in the ACEI/ARB group were matched to the nearest-neighbor controls at a 1:1 ratio based on propensity scores and not allowing replacement. A total of 404 patients were enrolled *via* propensity score matching, 202 of whom were treated with ACEI/ARB, while 202 were not. From PSM plot (Supplementary Figure 2), we could see that this model has achieved relatively good matching. No significant differences were observed among all included variates (*P* > 0.05). The primary endpoint, all-cause in-hospital mortality, was much higher in patients treated without ACEI/ARB (11.4 vs. 5.4%). The result remained consistent with the secondary endpoint, 28-day mortality (10.4 vs. 4.5%). And patients treated with ACEI/ARB showed a lower SOFA [3 (2–5) vs. 4 (2–6)] score though there was no statistically significance between these two groups. Besides, in our study, we found that patients with stage 3 hypertension had the highest mortality, and the most obvious clinical benefit could be obtained from the medication in this population (Supplementary Table 1).

**Table 3:** Baseline characteristics and clinical outcomes between patients treated with ACEI/ARB and those without ACEI/ARB after propensity score matching.

**Variables**	**ACEI/ARB (*n* = 202)**	**No-use of ACEI/ARB (*n* = 202)**	***P*-value**
Age (years)	69 (55–81)	67 (55–78)	0.185
Males [*n* (%)]	111 (55.0)	115 (56.9)	0.842
Heart rate (/min)	84 (73–94)	82 (72–94)	0.460
**Blood pressure**			
SBP (mmHg)	149 (143–161)	150 (142–159)	0.941
DBP (mmHg)	85 (69–93)	84 (71–96)	0.310
**Comorbidity**			
Congestive heart failure [*n* (%)]	46 (22.8)	42 (20.8)	0.631
coronary heart disease [*n* (%)]	35 (17.3)	41 (20.3)	0.525
Arrhythmia [*n* (%)]	61 (30.2)	54 (26.7)	0.441
Cardiac shock [*n* (%)]	3 (1.5)	4 (2.0)	1.000
COPD [*n* (%)]	15 (7.4)	18 (8.9)	0.717
Respiratory failure [*n* (%)]	43 (21.3)	50 (24.8)	0.479
Diabetes [*n* (%)]	70 (34.7)	70 (34.7)	1.000
Hypothyroidism [*n* (%)]	18 (8.9)	23 (11.4)	0.511
Cerebral hemorrhage [*n* (%)]	11 (5.4)	13 (6.4)	0.834
Cerebral infarction [*n* (%)]	9 (4.5)	9 (4.5)	1.000
AKI [*n* (%)]	63 (31.2)	58 (28.7)	0.588
CKD [*n* (%)]	42 (20.8)	46 (22.8)	0.718
Lymphoma [*n* (%)]	3 (1.5)	1 (0.5)	0.371
Anemia [*n* (%)]	37 (18.3)	31 (15.3)	0.427
**Laboratory events**			
WBC (K/uL)	10.4 (8.2–13.5)	9.9 (7.9–12.7)	0.131
BUN (mg/dL)	18.9 (12.6–28.1)	18.0 (12.0–29.2)	0.537
RBC (m/uL)	3.6 (3.1–4.2)	3.7 (3.2–4.2)	0.652
Hemoglobin (g/dL)	10.6 (9.5–12.7)	11.3 (9.6–12.4)	0.449
Creatinine (mg/dL)	0.9 (0.7–1.3)	1.0 (0.8–1.4)	0.460
PLT (K/uL)	200 (157–261)	200 (156–259)	0.703
Sodium (mmol/L)	139 (137–141)	139 (136–141)	0.631
Potassium (mmol/L)	4.1 (3.8–4.3)	4.0 (3.9–4.4)	0.845
Calcium (mmol/L)	8.6 (8.1–7.0)	8.5 (8.2–8.9)	0.609
Bicarbonate (mmol/L)	24.0 (22.0–26.0)	24.3 (22.0–26.8)	0.368
Chloride (mmol/L)	104 (101–107)	104 (99–107)	0.176
Glucose (mg/dL)	127 (111–151)	129 (108–160)	0.987
**Drugs**			
β blocker [*n* (%)]	113 (55.9)	109 (54.0)	0.620
Calcium channel blocker [*n* (%)]	49 (24.3)	50 (24.8)	1.000
Diuretics [*n* (%)]	60 (29.7)	67 (33.2)	0.522
Intravenous [*n* (%)]	15 (7.4)	18 (9.0)	0.717
**Outcomes**			
Hospital mortality [*n* (%)]	11 (5.4)	23 (11.4)	0.034
28-day mortality [*n* (%)]	9 (4.5)	21 (10.4)	0.025
SOFA max	3 (2–5)	4 (2–6)	0.352

### Development of ML Models and Model Explainability

**Figure 3:**
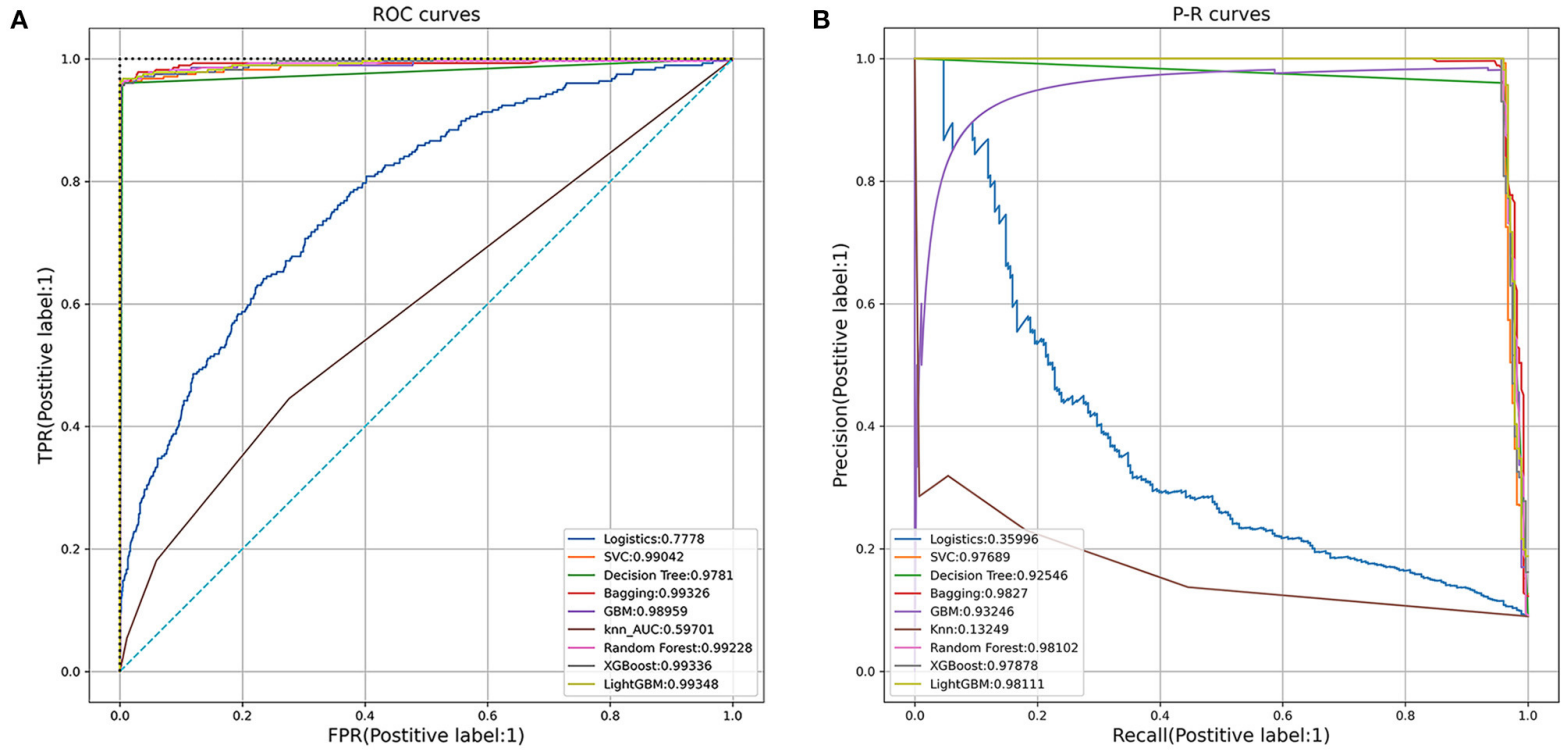
**(A)** Receiver operating characteristic (ROC) curves of the nine models. **(B)** Precision-Recall (P-R) curves of the nine models.

The logistic regression, support vector machine, decision tree, bagging, GBM, KNN, random forest, XGBoost and LightGBM models were established with a sample of 80% of the cohort generated randomly using a seed. The AUCs of the testing set were 0.7778, 0.9904, 0.9781, 0.9933, 0.9896, 0.5970, 0.9923, 0.9934, 0.9934 for each model, respectively (Figure 3A). Each model was validated by 5-fold cross-validation and the average accuracy was calculated to ensure the robustness of the result. The P-R curve was drawn to further compare the performance of different models (Figure 3B). The accuracy, precision and AUCs of nine models are summarized in Table 4. Among them, LightGBM performed best with the highest receiver operating characteristics (ROC) as well as highest accuracy and precision. Thus, we use LightGBM to build our final prediction model after hyperparameter optimization based on Grid and random hyper-parameter search. The hyper-parameters applied in the final LightGBM model were as follows after hyperparameter optimization: num_leaves = 31, learning_rate = 0.1, n_estimators = 300, max_depth = 2, colsample_bytree = 0.7. The AUC of the final model = 0.9025 (Supplementary Figure 3).

**Table 4:** Comparisons of nine different machine learning models.

**Model**	**Accuracy**	**Average precision**	**AUC**
Logistics regression	0.909	0.3600	0.7778
SVC	0.900	0.9769	0.9904
Decision tree	0.992	0.9255	0.9781
Bagging	0.916	0.9827	0.9933
GBM	0.995	0.9325	0.9896
KNN	0.895	0.1325	0.5970
Random tree	0.996	0.9810	0.9923
XGBoost	0.996	0.9788	0.9934
LightGBM	0.996	0.9811	0.9935

**Figure 4:**
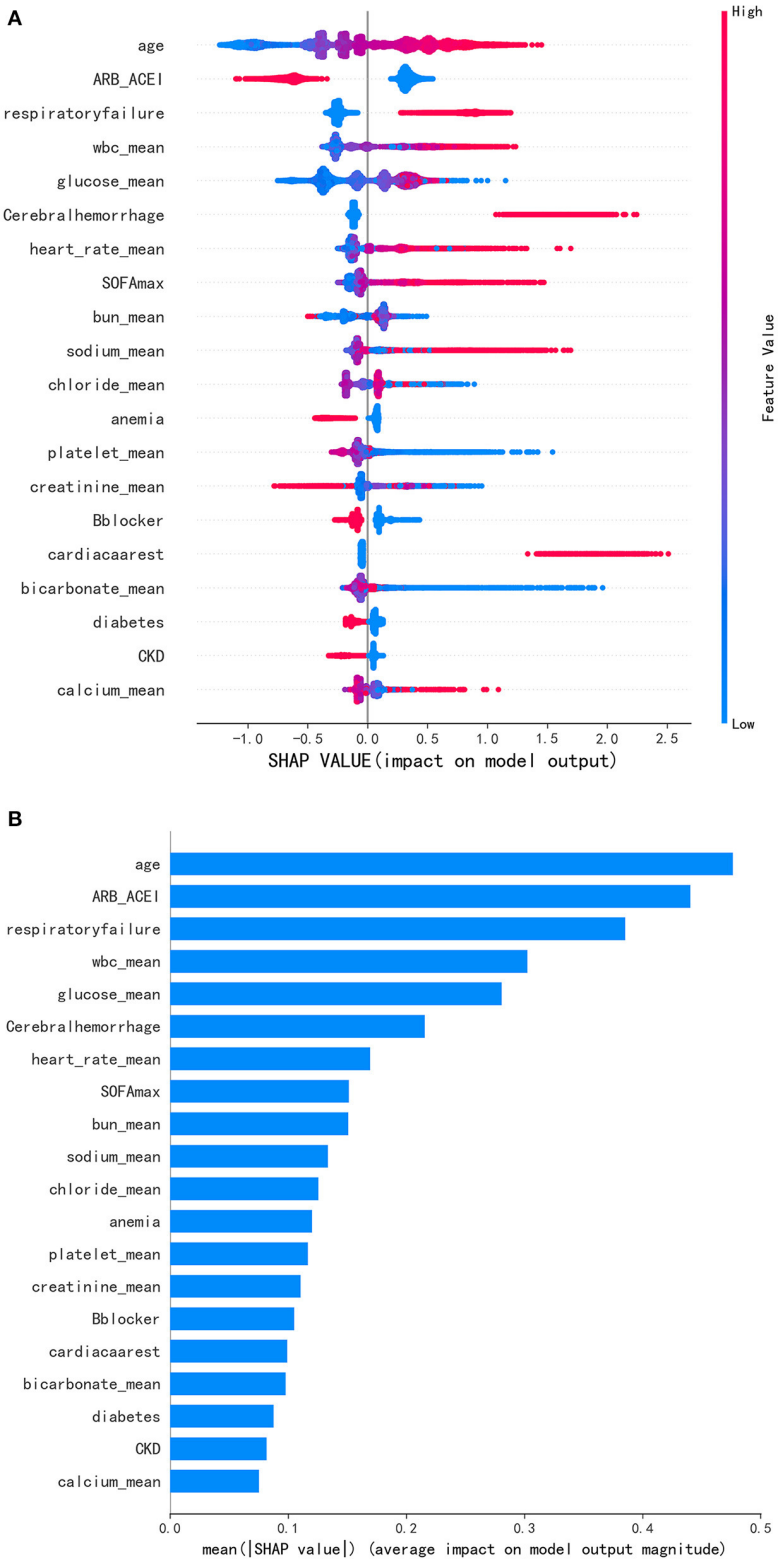
**(A)** Distribution of the impact each feature had on the full model output using SHapley Additive exPlanations (SHAP) values. **(B)** Significance of the predictors in the LightGBM model. Features were ranked according to the sum of absolute SHAP values of all samples. Longer bars indicated greater feature importance. Blue represented the lower value and red represented higher value of a single sample. A positive SHAP value represented an increase in the risk of hospital mortality.

The Shapley additive explanation (SHAP) was used in this study to make the model interpretable as shown in Figure 4. Features were ranked according to the sum of absolute SHAP values of all samples. Longer bars indicated greater feature importance. The top 20 most important features are listed in the figure. Blue represented the lower value and red represented higher value of a single sample. A positive SHAP value represented an increase in the risk of hospital mortality. As shown, age was the most important feature for the prediction of hospital mortality in patients with hypertension and the ACEI/ARB use ranked second. The absence of ACEI/ARB would significantly increase the hospital mortality of patients.

### Subgroup Analysis of Different Comorbidities

After being stratified by different types of comorbidities, as presented in Table 5, estimates of the association between ACEI/ARB use and hospital mortality were broadly consistent. In patients with coronary heart disease, congestive heart failure, cardiac shock, diabetes, cerebral hemorrhage and AKI, estimates of ORs were significantly lower than those without these comorbidities. In patients with respiratory failure and arrhythmia, estimates of ORs were significantly higher than those without these comorbidities. There were no statistically significant differences in patients with COPD, valvular disease, hypothyroidism, CKD. Patients who took β-blockers had higher OR than those who did not, which was not observed in patients using CCB, diuretics and intravenous antihypertensive drugs.

**Table 5:** Association of ACEI/ARB use and hospital mortality stratified by typed of comorbidities and drugs.

**Subgroups**	***N***	**Odds ratio (OR)**		***P*-value for interaction**
		**ACEI/ARB not use**	**ACEI/ARB use**	
Coronary heart disease				0.001
Yes	3,641	Ref	0.22 (0.16–0.29)	
No	11,711	Ref	0.31 (0.26–0.37)	
Congestive heart failure				0.02
Yes	3,012	Ref	0.25 (0.19–0.33)	
No	12,340	Ref	0.29 (0.24–0.34)	
Arrthymias				<0.001
Yes	3,684	Ref	0.28 (0.22–0.35)	
No	11,668	Ref	0.27 (0.22–0.33)	
Cardiac shock				<0.001
Yes	251	Ref	0.15 (0.08–0.29)	
No	15,101	Ref	0.29 (0.24–0.33)	
COPD				0.521
Yes	1,249	Ref	0.26 (0.16–0.45)	
No	14,103	Ref	0.29 (0.24–0.34)	
Valvular disease				0.105
Yes	1,529	Ref	0.28 (0.19–0.41)	
No	13,823	Ref	0.28 (0.24–0.33)	
Respiratory failure				<0.001
Yes	3,481	Ref	0.31 (0.26–0.39)	
No	11,871	Ref	0.27 (0.22–0.34)	
Diabetes				0.035
Yes	4,721	Ref	0.25 (0.19–0.32)	
No	10,631	Ref	0.30 (0.25–0.36)	
Hypothyroidism				0.855
Yes	1,915	Ref	0.33 (0.22–0.48)	
No	13,437	Ref	0.28 (0.24–0.33)	
Cerebral hemorrhage				<0.001
Yes	1,042	Ref	0.21 (0.15–0.29)	
No	14,310	Ref	0.27 (0.22–0.32)	
AKI				<0.001
Yes	3,980	Ref	0.22 (0.17–0.28)	
No	11,372	Ref	0.35 (0.28–0.40)	
CKD				0.227
Yes	2,929	Ref	0.29 (0.21–0.40)	
No	12,423	Ref	0.28 (0.24–0.33)	
CCB				0.529
Yes	3,490	Ref	0.31 (0.24–0.41)	
No	11,822	Ref	0.26 (0.22–0.36)	
β blocker				0.006
Yes	7,542	Ref	0.34 (0.28–0.42)	
No	7,810	Ref	0.23 (0.18–0.30)	
Diuretics				0.900
Yes	4,671	Ref	0.28 (0.23–0.36)	
No	10,681	Ref	0.26 (0.21–0.32)	
Intravenous drugs				0.649
Yes	1,503	Ref	0.27 (0.17–0.43)	
No	13,849	Ref	0.29 (0.25–0.34)	

## Discussion

This study selected patients who were admitted to ICU with hypertension as study participants and explored the associations between the use of ACEI/ARB and clinical outcomes. Data from this large accessible clinical database were analyzed retrospectively. The results indicated that the use of ACEI/ARB was a significant protective factor for critically ill patients with hypertension that decreased hospital mortality and 28-day mortality. Different machine learning models, including propensity score matching, were employed to adjust potential confounders and the results remained consistent. Besides, we found that the use of ACEI/ARB in patients with severe hypertension (stage ≥ 2) could significantly reduce mortality compared to stage 1. The ICU LOS and hospital LOS were close between the two groups, while the SOFA score in patients treated with ACEI/ARB was lower than those treated without ACEI/ARB. These results are consistent in patients with or without acute or chronic kidney injury, notably, patients with AKI even benefit more from ACEI/ARB use. A previous propensity score-matched cohort study investigated the association between the use of ACEI/ARB prior to ICU admission and in-hospital mortality (16) and found that patients treated with ACEI/ARB prior to ICU had a lower mortality (12.6 vs. 22.1%), which was consistent with clinical outcomes from this analysis. Some patients in our analysis might also take ACEI/ARB prior to ICU, our results thus confirmed their study results in some way.

The research method of machine learning is increasingly used in the medical field. It not only provides a new method in the prediction model (17), but also shows prominence in some classification problems (18). The novelty of this study is that through the sophisticated ML model and SHAP method, 20 important features closely associated with hospital mortality were identified and results demonstrated that ACEI/ARB use ranked second among them.

Several studies have demonstrated that hypertension was a risk factor associated with the severity of COVID-19 and high ICU mortality in some reports (19, 20). Geng et al. reached the same conclusion and further found that hypertensive patients during ICU hospitalization with stage 3 hypertension had the highest fatality compared with stage1 and stage2 hypertensive patients (21). And stage 3 hypertension was an independent risk factor in multivariate Cox regression model with HR = 1.26 (95% CI = 1.02–1.56). All these studies have demonstrated that hypertension was one of the independent risk factors for fatality in critically ill patients. Thus, controlling hypertension in these patients was rather effective to reduce hospital mortality.

There has been an ongoing debate about whether ACEI/ARB medication should be employed in patients with renal insufficiency. As a clinical consensus, ACEI drugs should be treated with caution in patients with elevated creatinine. A previous study demonstrated that a higher proportion of patients with cardiorenal comorbidity had creatinine increases of 30% or more, which resulted in unsatisfactory outcomes (13). However, another study of AKI patients found that ACEI/ARB medication in these patients appeared to be associated with lower mortality but a higher risk of hospitalization for a renal cause, thus indicating a potential benefit of ACEI or ARB use after AKI, but cautious monitoring for renal-specific complications may be warranted (22). In a recent study, researchers found that longer predialysis ACEI/ARB exposure was associated with lower postdialysis mortality (23). In the subgroup analysis of this study, we found that patients with AKI might benefit more from ACEI/ARB use, and patients with CKD shared similar benefit from ACEI/ARB use in these patients, the results clearly denied the negative impact of ACEI/ARB use for outcome due to ACEI/ARB-induced increased creatinine levels.

Coronavirus disease (COVID)-19 was a heterogeneous condition and was characterized by interstitial pneumonia that could lead to impaired gas-exchange, acute respiratory failure and death (24). From a systematic review and meta-analysis, ACEI/ARB exposure was associated with a lower risk of mortality compared to those treated with non-ACEI/ARB antihypertensive drugs (OR = 0.48, 95% CI, 0.29–0.81; *P* = 0.006) (25). Notably, Zhang et al. found that among hospitalized patients with COVID-19 and coexisting hypertension, inpatient use of ACEI/ARB was associated with a lower risk of all-cause mortality than ACEI/ARB non-users (26). Interestingly, respiratory failure was a significant risk factor for hospital mortality in our final model and ranked third (Figure 4). Respiratory failure was common comorbidity in critically ill patients, accounting for 22.7% in our study participants. In the subgroup analysis, we found that ACEI/ARB still had a strong protective effect on patients with respiratory failure (OR = 0.31). Therefore, for patients with severe lung disease complicated with respiratory failure, the use of ACEI/ARB might have a positive impact on the prognosis.

In addition to ACEI/ARB, β-blockers, CCB, diuretics and intravenous antihypertensive drugs were commonly used drugs in patients with hypertension. Researchers have found that β-blockers could reduce blood pressure by reducing oxidative stress and endothelial dysfunction (27). However, current American guidelines (Joint National Committee VIII) considered β-blockers as a third therapeutic choice for treating hypertension and consistently, NICE guidelines stated that β-blockers were not preferred initial treatment (28, 29). This study also revealed that the use of β-blockers was an important protective factor in our study participants and its use could reduce hospital mortality (Figure 4). Recently, a study suggested that CCB administration to COVID-19 patients with hypertension as comorbidity might improve the disease outcome (30). However, in our study we didn't find that CCB was a protective factor for critically ill patients with hypertension. In this case, we think it may be due to the negative inotropic effect on the myocardium. Notably, the protective effect of ACEI/ARB was significantly lower in patients who used β-blockers than those who did not in the subgroup analysis [OR, 0.34 (0.28–0.42) vs. 0.23 (0.18–0.30)] while we did not observe the interaction between ACEI/ARB and CCB, diuretics, intravenous antihypertensive. In the PSM analysis, we matched the β-blocker, CCB, diuretics and intravenous antihypertensive drugs between the two groups and the effect of ACEI/ARB on the reduction of mortality remained stable.

The cerebral perfusion pressure was manipulated together by mean arterial blood pressure and intracranial pressure, Cocchi and Edlow et al. suggested that the optimal blood pressure targets in the setting of acute ischemic stroke, acute subarachnoid hemorrhage, and spontaneous intracerebral hemorrhage remained somewhat controversial (31). Nicardipine or Clevidipine was commonly the first choice in patients with acute ischemic disease of cerebrovascular disease to lower high blood pressure. In this study, critically ill patients with comorbidity cerebral hemorrhage and infarction were included with 6.8 and 3.6%, retrospectively. This study laid a foundation of using ACEI/ARB in such patients, which required more clinical evidence and further randomized controlled trials.

Interestingly, ACEI/ARB are also frequently used in patients with heart failure and coronary heart disease (32). In the subgroup analysis, we also found that ACEI/ARB played a better protective role in patients with coronary heart disease and heart failure with decreased ORs (as presented in Table 5). This result lays a foundation for the routine use of these drugs in patients with severe cardiovascular disease.

As for the length of stay in hospital and ICU, we found that there was a statistically significant difference between the two groups, though the data between the two groups were close. The LOS of hospital in patients treated with ACEI/ARB was 6.58 (3.88–10.86) and LOS of ICU was 1.92 (1.06–3.73), which were longer than those in patients treated without ACEI/ARB. For this result, we believe that it may be due to a higher mortality rate in patients without medication and more patients died in the early stages of hospitalization, resulting in shorter LOS. The severity of the disease in the treatment group was lower than that in the other group for the sofa was 3 (1–5) after the use of ACEI/ARB compared to 4 (2–6).

There are some limitations of the study. It is challenging work to analyze such a large heterogeneous population. This is a single-center retrospective observational study and selection bias was inevitable, though we employed a propensity score matching to adjust confounders. Therefore, a randomized controlled prospective study is needed to validate results from this analysis. Besides, our secondary endpoint was 28-day mortality, which could not completely reflect the long-term mortality and outcome. Additionally, some patients may be unable to take medicine because of their critical condition, which needs to be taken into consideration. In this study, the relationship between drug dose, medication time and mortality were not referred, which needs further investigation.

## Conclusion

The use of ACEI/ARB in critically ill patients with hypertension during ICU stay is related to lower all-cause in-hospital mortality, independent of renal dysfunction.

## Data Availability Statement

The raw data supporting the conclusions of this article will be made available by the authors, without undue reservation.

## Ethics Statement

Ethical review and approval was not required for the study on human participants in accordance with the local legislation and institutional requirements. Written informed consent for participation was not required for this study in accordance with the national legislation and the institutional requirements.

## Author Contributions

BY conceived the theme and wrote the manuscript. SX edited the code. CS and DW improved the manuscript. YC and ZZ optimized the image. All authors contributed to manuscript revision and approved the submitted version.

## Funding

CS was supported by the National Natural Science Foundation of China (81871105) and Shanghai Shenkang Hospital Development Center (SHDC2020CR1042B).

## Conflict of Interest

The authors declare that the research was conducted in the absence of any commercial or financial relationships that could be construed as a potential conflict of interest.

## Publisher's Note

All claims expressed in this article are solely those of the authors and do not necessarily represent those of their affiliated organizations, or those of the publisher, the editors and the reviewers. Any product that may be evaluated in this article, or claim that may be made by its manufacturer, is not guaranteed or endorsed by the publisher.

## Abbreviations

ACEI/ARB: angiotensin-converting enzyme inhibitor/angiotensin II receptor blocker
ICU: intensive care units
SBP: systolic blood pressure
DBP: diastolic blood pressure
COPD: chronic obstructive pulmonary disease
AKI: acute kidney injury
CKD: chronic kidney disease
WBC: white blood cell
BUN: blood urea nitrogen
RBC: red blood cell
PLT: platelets
LOS: length of stay
SOFA: sequential organ failure assessment
OR: odds ratio
CCB: calcium channel blocker
ML: machine learning
AUC: area under the curve.
