# Supplementary material for: ACEI/ARB Medication During ICU Stay Decrease All-Cause In-hospital Mortality in Critically Ill Patients With Hypertension: A Retrospective Cohort Study Based on Machine Learning
Source: Front Cardiovasc Med. 2022 Jan 12;8:787740. doi: 10.3389/fcvm.2021.787740 (PMC8791359; doi:10.3389/fcvm.2021.787740)
Supplement: Supplementary file 1 [file Table_1.DOC]

**Table 1 association between hypertension stage and clinical outcomes**

| variables | Stage1 | | Stage2 | | Stage3 | | P-value |
| --- | --- | --- | --- | --- | --- | --- | --- |
|  | use | not use | use | not use | use | not use |  |
| Numbers(n(%)) | 3047(31.4%) | 6657(68.6%) | 1369(37.8%) | 2241(62.2%) | 786(38.3%) | 1267(61.7%) |  |
| Hospital mortality(n(%)) | 113(3.7%) | 748(11.2%) | 62(4.6%) | 315(14.1%) | 30(3.8%) | 224(17.7%) | ＜0.001 |
| 28-day mortality(n(%)) | 103(3.4%) | 714(10.7%) | 56(4.1%) | 304(13.6%) | 30(3.8%) | 218(17.2%) | ＜0.001 |
