# Supplementary figures and images for: ACEI/ARB Medication During ICU Stay Decrease All-Cause In-hospital Mortality in Critically Ill Patients With Hypertension: A Retrospective Cohort Study Based on Machine Learning
Source: Front Cardiovasc Med. 2022 Jan 12;8:787740. doi: 10.3389/fcvm.2021.787740 (PMC8791359; doi:10.3389/fcvm.2021.787740)

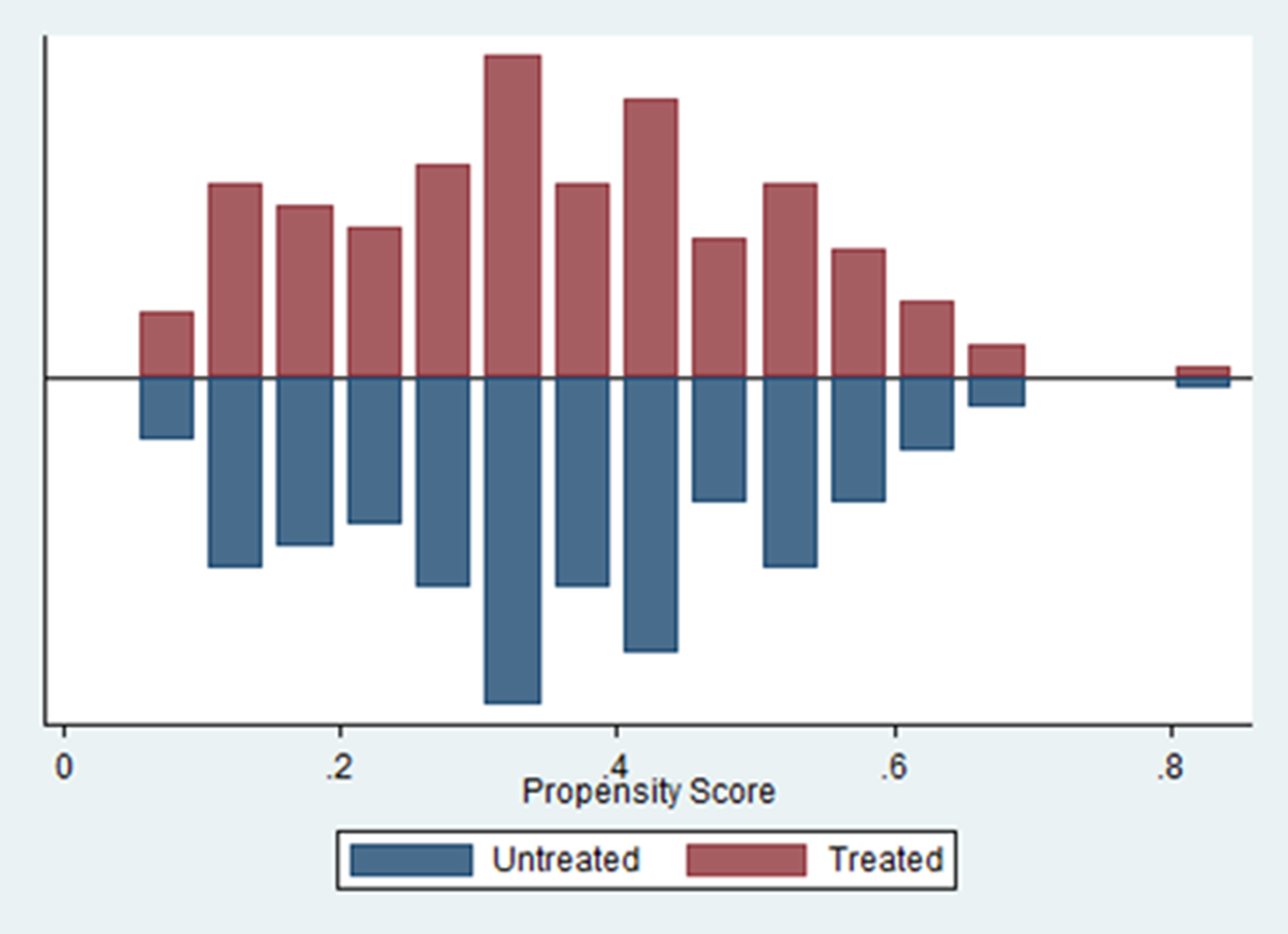

Supplement: Supplementary file 2 [file Image_1.JPG]

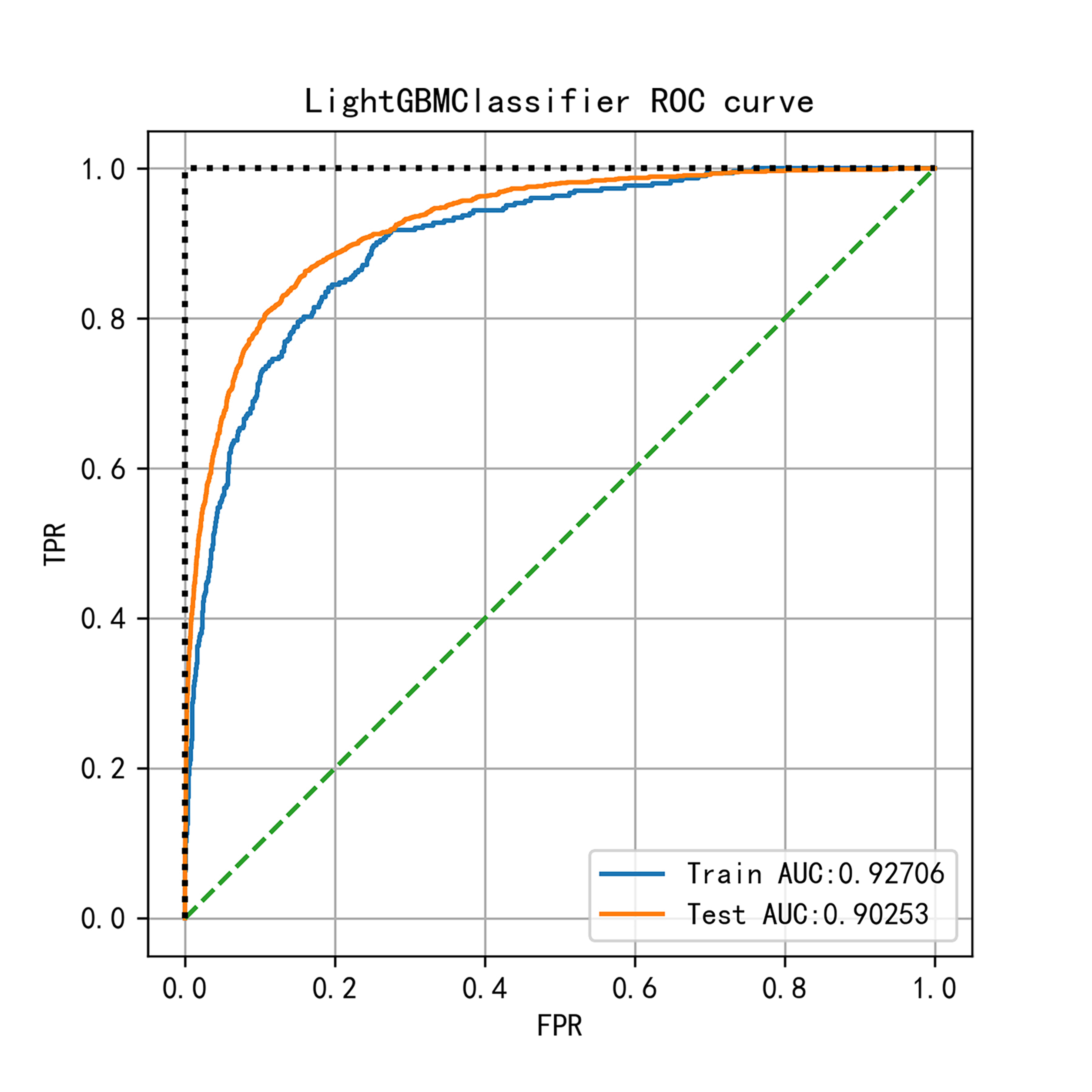

Supplement: Supplementary file 3 [file Image_2.JPG]
